# Variation at *GRN* 3′-UTR rs5848 Is Not Associated with a Risk of Frontotemporal Lobar Degeneration in Dutch Population

**DOI:** 10.1371/journal.pone.0007494

**Published:** 2009-10-22

**Authors:** Javier Simón-Sánchez, Harro Seelaar, Zoltán Bochdanovits, Dorly J. H. Deeg, John C. van Swieten, Peter Heutink

**Affiliations:** 1 Department of Clinical Genetics, Section of Medical Genomics, VU University Medical Centre, Amsterdam, The Netherlands; 2 Department of Neurology, Erasmus Medical Center, Rotterdam, The Netherlands; 3 Longitudinal Aging Study Amsterdam, EMGO Institute for Health and Care Research, VU University Medical Centre, Amsterdam, The Netherlands; University of Muenster, Germany

## Abstract

**Background:**

A single nucleotide polymorphism (rs5848) located in the 3′- untranslated region of *GRN* has recently been associated with a risk of frontotemporal lobar degeneration (FTLD) in North American population particularly in pathologically confirmed cases with neural inclusions immunoreactive for ubiquitin and TAR DNA-binding protein 43 (TDP-43), but negative for tau and alpha-synuclein (FTLD-TDP).

**Methodology/Principal Findings:**

In an effort to replicate these results in a different population, rs5848 was genotyped in 256 FTLD cases and 1695 controls from the Netherlands. Single SNP gender-adjusted logistic regression analysis revealed no significant association between variation at rs5848 and FTLD. Fisher's exact test, failed to find any significant association between rs5848 and a subset of 23 pathology confirmed FTLD-TDP cases.

**Conclusions/Significance:**

The evidence presented here suggests that variation at rs5848 does not contribute to the etiology of FTLD in the Dutch population.

## Introduction

Frontotemporal lobar degeneration (FTLD) is the second most common form of presenile dementia after Alzheimer's disease (AD), representing ∼5% of all dementia cases and as much as 10–20% of presenile dementia [1], [2].

FTLD is characterized by not specific symptoms such as apathy and lack of interest, usually followed by specific features such as disinhibition or progressive language loss. These symptoms gradually evolve to cognitive impairment and dementia.

Pathologically, FTLD is characterized by neural loss and gliosis in the cortical lamina II of the frontal and temporal lobes. FTLD is pathologically heterogeneous and can be divided in FTLD with tau-positive inclusions (FTLD-tau), and FTLD with neural inclusions immunoreactive for ubiquitin and TAR DNA-binding protein 43 (TDP-43), but negative for tau and alpha-synuclein (FTLD-TDP) [3], [4]. In some FTLD-TDP cases, these pathological features co-occur with motor neuron disease (FTLD-MND) [5], which is characterized by the degeneration of upper and/or lower motor neurons.

FTLD has a high familial incidence with up to 50% of patients reported to have a family history of similar dementia, parkinsonism or MND [6], [7], [8], [9]. It has been reported that loss of function mutations in the gene encoding the secreted growth factor progranulin (*GRN*) are a major cause of FTLD-TDP worldwide counting for as much as 25% of familiar FTLD-TDP [10], [11], [12].

Recently, Rademakers and colleagues demonstrated that a single nucleotide polymorphism (rs5848) located in the 3′- untranslated region (3′-UTR) of *GRN* is significantly associated with the development of FTLD, particularly in a homogeneous series of patients with a neuropathological diagnosis of FTLD-TDP [13]. They showed that homozygous carriers of the risk T-allele have a 3.2-fold increased risk to develop FTLD-TDP compared with homozygous carriers for the major C-allele. This risk was increased when excluding those patients in which MND had been diagnosed. Moreover, since rs5848 is located in the predicted binding site of miRNA miR-659 and that these small RNAs are known to posttranscriptionally modulate expression of target mRNAs, the authors hypothesized that rs5848 increases the risk of FTLD-TDP by altering the miRNA regulation of *GRN*. They successfully demonstrate that miR-659 binds more efficiently to the GRN 3′-UTR containing the risk T-allele of rs5848 resulting in a drop of GRN protein levels. As expected, no changes were detected in the GRN mRNA levels.

In an effort to replicate the results obtained by Rademakers and collaborators and to confirm the role of rs5848 in modulating the risk for FTLD, this SNP was genotyped in a large series of 287 FTLD cases (before quality control procedures) and 1695 controls from the Netherlands [14], [15].

## Methods

### Participants

Approval was given by the Medical Ethical Committee of the Erasmus University, Rotterdam, the Netherlands; after written informed consent of the patients that their information could be stored in the hospital database and used for research.

A total of 287 patients with FTD were ascertained from an ongoing genetic-epidemiologic study conducted in the Netherlands since 1994 after referral to the out-clinic department of the Erasmus Medical Center, or after visiting nursing homes and psychogeriatric hospitals by the research physician [8], [15]. Detailed clinical history was obtained from the spouses and first-degree relatives using a checklist of behavioral and cognitive changes, and motor neuron signs.

The age at onset was defined as the age at which the first symptom, compatible with FTD diagnosis, was observed by a close relative or caretaker. During the neurological examination carried out in all patients, special attention was paid to the presence of extrapyramidal and motor neuron disease signs.

After genotyping, all samples presenting with mutations in *MAPT* and *GRN* genes, those in which an autopsy did not confirm FTLD pathology, those without a Caucasian ancestry and those closely related to some other sample in our dataset, where removed from further analysis. Thus, our final FTLD cohort consisted of 256 cases of which 120 where males and 136 females. The main age at onset was 57.73 years ranging from 28.64 to 75.95 years (SD = 8.94).

Control samples consisted of 805 males and 890 females from the Longitudinal Aging Study Amsterdam [17](LASA, http://www.lasa-vu.nl/) with a mean age of 68.78 years at the time of medical examination (range: 54.80 – 88.77, SD = 9.40 years).

Although we are aware that the age distribution of this cohort cannot be matched to that from the FTLD cases, a single SNP sex-adjusted linear regression analysis in these samples considering the age at examination as a quantitative phenotype showed that genotypes at rs5848 are equally distributed across age (*P_genotypic_* = 0.29).

### Genotyping

Custom TaqMan® MGB probes and primers from Applied Biosystems (www.appliedbiosystems.com) were utilized to genotype rs5848 in all samples previously described. Primer and probes were design with the File Builder v3.1 software (www.appliedbiosystems.com) and genotyping was performed in a LightCycler 480 II PCR system using LightCycler 480 Probes Master mix (www.roche.com) as per manufacturer's instructions. For each reaction, 20 ng of genomic DNA was utilized.

### Statistical analysis

All tests and estimates were performed using the PLINK toolset for whole-genome analysis (http://pngu.mgh.harvard.edu/~purcell/plink) [18].

Genotyping information at rs5848 was tested for departures from the Hardy-Weinberg equilibrium.

Single SNP gender-adjusted logistic regression analysis was performed to test for association between rs5848 and FTLD using a recessive model. Besides, to test for the effect of rs5848 in FTLD-TDP confirmed cases, a Fisher's exact test for association was performed.

Statistical significance for differences in age at onset was tested using a gender-adjusted linear regression analysis and considering age at onset as a quantitative trait. Before performing this analysis, R v.2.7.2 software (http://cran.r-project.org/) revealed a normal distribution of the data.

## Results

In order to confirm the results obtained by Rademakers and collaborators who described an association between rs5848 and a risk for FTLD, especially in a subset of pathologically confirmed FTLD-TDP patients [13], we undertook a logistic regression analysis of variation at this *locus* in a large series of FTLD cases and controls from the Netherlands.

After removing samples with mutations in *MAPT* or *GRN* genes, those not showing a clear Caucasian ancestry, those in which an autopsy could not confirm the existence of FTLD and those closely related to some other individual in our FTLD cohort, our final dataset consisted of 256 FTLD cases and 1695 controls. There were no departures from Hardy-Weinberg equilibrium.

Given that the association described by Rademakers' and colleagues at rs5848 was because of an increase of TT genotype frequency in patients compared with controls, we decided to perform a single SNP gender-adjusted logistic regression following a recessive model of association. Results derived from this analysis revealed no significant association between rs5848 and FTLD (*P_recessive_* = 0.57; table 1).

**Table 1:** Genotype frequencies and association results for the tests performed in the Dutch series.

Samples used	Genotype frequencies (cases/controls)	Test applied	*p* value	OR (95% c.i)
	CC: 49.79%/49.69%			
Entire cohort (256 cases/1645 controls)	CT: 39.33%/40.61%	Logistic regression	*P_recessive_* = 0.57	1.14 (0.73–1.77)
	TT: 10.87%/9.69%			
	CC: 54.54%/49.69%			
FTLD-TDP confirmed (23 cases/1645 controls)	CT: 36.36%/40.61%	Fisher's exact test	*P_Fisher_* = 0.74	0.87 (0.45–1.70)
	TT: 9.09%/9.69%			
	CC: 52.94%/49.69%			
FTLD-TDP confirmed/MND excluded (18 cases/1645 controls)	CT: 41.17%/40.61%	Fisher's exact test	*P_Fisher_* = 0.71	0.84 (0.39–1.80)
	TT: 5.88%/9.69%			

In order to confirm Rademaker's and colleagues' results in which they focused their analyses on a homogenous series of patients with confirmed TDP-43-positive neuronal inclusions, Fisher's exact test of association was performed in a subset of our series of 23 pathologically confirmed FTLD-TDP cases. Besides, in an effort to replicate Rademakers' results showing that the effect detected was increased when individuals with confirmed MND pathology were removed, Fisher's exact test was repeated after the removal of 5 patients with a confirmed MND pathology. Results derived from these analyses failed to detect any association between rs5848 and FTLD (table 1).

Finally, in an effort to test whether rs5848 may have an influence on the age at onset of FTLD in our cohort, a gender-adjusted linear regression analysis following a recessive model was performed considering age at onset as an alternative quantitative phenotype. No association was detected after this analysis in the entire cases cohort (*P_recessive_* = 0.50).

## Discussion

Here we present the analysis of rs5848 in a large cohort of FTLD cases and controls from the Netherlands. Variation at this *locus* was previously associated with a risk of FTLD in a large series of 339 individuals from three different subgroups of cases (Olmsted County community-based series, ADRC-FTLD referral series and the tertiary referral series) and 934 controls ascertained through the Mayo Clinic at Jacksonville and Scottsdale [13], [19]. The reported association was driven by an excess of homozygous patients for the T allele (*P*_genotypic_ = 0.002). Analysis of variation at this *locus* in our Dutch cohort showed no significant differences between the frequency of homozygous T carriers in cases versus controls (*P*_recessive_ = 0.57). Although we cannot rule out a minimal contribution of this *locus* on modeling a risk for FTLD, the sample size of the population utilized for our statistical tests (256 FTLD cases and 1645 controls) provides an adequate power to detect an effect of the magnitude originally described (OR = 2.9 for T/T carriers versus T/C and C/C carriers). Thus, the evidence presented here suggests that variation at rs5848 does not contribute to the etiology of FTLD in the Dutch population.

We also failed to replicate Rademakers and collaborators' results showing an association between rs5848 and a subset of neuropathologically confirmed FTLD-TDP cases from the Mayo Clinic brain bank [13]. Given the reduced number of FTLD-TDP confirmed cases in our series, this could be due to a type II error and thus, a role of rs5848 on modulating a risk for this pathological subtype of the disease cannot be discarded. However, the fact that no association was detected in the whole FTLD series suggests otherwise.

As proposed by Mackenzie *et al*., who established a classification scheme of FTLD-TDP cases based on the distribution of the neuronal cytoplasmatic inclusions, dystrophic neurites and the presence of neuronal intranuclear inclusions [20], our pathological series consisted on 8 type 2 and 15 type 3 patients. Although the lack of type 1 FTLD-FTD patients in our cohort could explain the discrepancies observed between ours and Rademakers' results, the increase of TT carriers among type 1 FTLD-TDP cases detected in the Mayo clinic series was not statistically significant after Fisher's exact test of association (*P* = 0.26).

Recently, Viswanathan and collaborators investigated the role of six different SNPs in the *GRN* gene (including rs5848) in a Finnish population with AD [21]. Although no association was found for either of the SNPs assayed, they suggest that variation in rs5848 and two other SNPs may increase the risk for developing AD in a gender-specific manner. Given the similarities between FTLD and AD, logistic regression models were adjusted for gender. However, this approach did not show any association between rs5848 and a risk of FTLD in our population.

In a similar report as that presented herein, Rollinson and collaborators also failed to find any association between rs5848 an FTLD in 3 different European populations [22]. Together with ours, these results suggest that rs5848 has no effect on modulating a risk for FTLD in European populations, being in discordance with those results obtained by Rademakers and colleagues in North American population. Although a type II error in both Rollinson's report and the data presented herein cannot be ruled out, the fact that the minor allele frequency at this *locus* ranges across populations from 0.16 to 0.77 (for HapMap-CEU and HapMap YRI respectively) shows the susceptibility of this SNP to be affected by case-control admixture and, thus, lead to type I errors. Thus, results observed by Rademakers' and collaborators may represent a slant in the selection of the FTLD-TDP patients.

In summary we conclude that variation at rs5848 does not affect the risk for developing FTLD in European populations. Although a small effect in the FTLD-TDP pathological subtype of the disease cannot be discarded, evidence presented here and elsewhere does not support this idea. Further genotyping of different populations of these and other characteristics is needed for better understanding the role of rs5848 on the pathogenesis of FTLD.
